# Multisystem Inflammatory Syndrome Following SARS-CoV-2 Infection in Children: One Year after the Onset of the Pandemic in a High-Incidence Area

**DOI:** 10.3390/v13102022

**Published:** 2021-10-07

**Authors:** Marianna Fabi, Emanuele Filice, Carlotta Biagi, Laura Andreozzi, Daniela Palleri, Bianca Elisa Mattesini, Alessia Rizzello, Liliana Gabrielli, Chiara Ghizzi, Daniela Di Luca, Fabio Caramelli, Alessandro De Fanti, Marcello Lanari

**Affiliations:** 1 Department of Pediatrics, Istituto di Ricovero e Cura a Carattere Scientifico St. Orsola Polyclinic, University of Bologna, 40138 Bologna, Italy; marianna.fabi@aosp.bo.it (M.F.); carlotta.biagi@aosp.bo.it (C.B.); laurandreozzi@gmail.com (L.A.); daniela.palleri@gmail.com (D.P.); bianca.mattesini@gmail.com (B.E.M.); alessia.rizzello2@studio.unibo.it (A.R.); marcello.lanari@unibo.it (M.L.); 2 Microbiology Unit, Istituto di Ricovero e Cura a Carattere Scientifico St. Orsola Polyclinic, University of Bologna, 40138 Bologna, Italy; liliana.gabrielli@aosp.bo.it; 3 Department of Pediatrics, Maggiore Hospital, 40133 Bologna, Italy; chiara.ghizzi@ausl.bologna.it; 4 Department of Anesthesiology, Istituto di Ricovero e Cura a Carattere Scientifico St. Orsola Polyclinic, University of Bologna, 40138 Bologna, Italy; daniela.diluca@aosp.bo.it (D.D.L.); fabio.caramelli@aosp.bo.it (F.C.); 5 Pediatrics Unit, Arcispedale Santa Maria Nuova, Via Risorgimento 80, 42123 Reggio Emilia, Italy; alessandro.defanti@gmail.com

**Keywords:** multisystem inflammatory syndrome in childhood, cytokine profiles, COVID-19, SARS-COV-2, Kawasaki Disease, cardiac involvement

## Abstract

SARS-CoV-2 infection in children can trigger cardiovascular manifestations potentially requiring an intensive treatment and defining a new entity named Multisystem Inflammatory Syndrome in Children (MIS-C), whose features partially overlap with Kawasaki Disease (KD). A cross-sectional study including all diagnoses of MIS-C and KD from April 2020 to May 2021 in our metropolitan area was conducted evaluating clinical, laboratory (including immunological response, cytokines, and markers of myocardial damage), and cardiac (coronary and non-coronary) features at onset of the diseases. Evolution of ventricular dysfunction, valve regurgitations, and coronary lesions was documented. The severity of the disease was also considered based on the need for inotropic support and ICU admission. Twenty-four MIS-C were diagnosed (14 boys, median age 82 months): 13/24 cases (54.17%) presented left ventricular dysfunction, 12/24 (50%) required inotropic support, and 10/24 (41.67%) developed coronary anomalies (CALs). All patients received steroids and IVIG at a median time of 5 days (IQR1:4, IQR3:6.5) from onset of fever and heart function normalized 6 days (IQR1: 5, IQR3: 7) after therapy, while CALs persisted in one. One patient (12.5%) required infliximab because of refractory disease and still presented CALs 18 days after therapy. During the same study period, 15 KD were diagnosed: none had ventricular dysfunction, while 7/15 (46.67%) developed CALs. Three out of 15 patients (20%) still presented CALs 46 days from onset. Compared to KD, MIS-C pts have significantly higher IL8 and similar lymphocytes subpopulations. Despite a more severe presentation and initial cardiac findings compared to KD, the myocardial injury in MIS-C has a rapid response to immunomodulatory treatment (median time 6 days), in terms of ventricular function, valve regurgitations, and troponin. Incidence of CALs is similar at onset, but it tends to regress in most of the cases of MIS-C differently than in KD where CALs persist in up to 40% in the subacute stage after treatment.

## 1. Introduction

COVID-19 pandemic has severely affected 2020, rapidly becoming a challenging global health issue. 

SARS-CoV-2 is the causative pathogen of the COVID-19 pandemic and belongs to the beta-coronaviruses group. The positive-sense single-stranded RNA virus has an enveloped capsid that expresses the spike protein, which is a critical protein involved in its pathogenesis [1]. 

The virus enters cells through binding to the angiotensin-converting enzyme 2 (ACE2) receptor, which is widely distributed over a multitude of organs and contributes to the systemic nature of the disease [2,3,4].

Despite firstly considered as an adult condition [5,6], growing evidence has led to describing a new syndrome developing 3–6 weeks following SARS-CoV-2 infection and sharing clinical features with Kawasaki Disease (KD) [7,8,9], which is named Multisystem Inflammatory Syndrome in Children (MIS-C) [10].

Different diagnostic criteria have been released by scientific societies [11,12,13], all agreeing that MIS-C is to be intended as an infectious-related autoimmunity syndrome unleashed by SARS-CoV-2 occasionally leading to dramatic clinical patterns. 

MIS-C and KD share clinical manifestations such as persistent fever, skin rash, conjunctival hyperemia, mucosal changes, gastrointestinal symptoms, and cardiac involvement. Coronary artery lesions (CALs), myocardial dysfunction, and hypotension or shock can be present in both MIS-C and KD. The exact mechanism of myocardial damage could be related to both direct and indirect injury. Autoptic studies on patients deceased after developing MIS-C identified SARS-CoV-2 in heart tissue even in the absence of significant heart inflammation [14]. The cytokine storm induced by the virus [15] contributes to depressed myocardial function, often requiring inotropic support in an intensive care setting [16,17].

COVID-19 in adults is known to be characterized by endothelial dysfunction, whose role has largely been debated in KD [18]. Authors hypothesized endothelial dysfunction among the actors of KD pathogenesis [19], proposing it as a potential accelerator of atherosclerosis’ process [20,21]. The European Society of Cardiology considered Kawasaki-like vasculitis involvement in children as a strong suggestion for endothelial dysfunction in COVID-19 patients [22].

Endothelial involvement in MIS-C results in the development of dilations and aneurysms in coronary arteries and systemic vasoplegia. Autoptic analysis of endothelial cells in MIS-C revealed viral particles, disruption of capillary walls, and fibrin clot formation [14]. These findings, although not surprising since ACE2 is highly expressed on coronaries vascular endothelium [3], provide a partial explanation of MIS-C pathogenesis. 

The young age of affected subjects, the epidemics and the self-limited nature of illness, the upregulation of interferon response genes in lung and coronary arteries [23,24], the identification of virus-like particles in inclusion bodies in ciliated bronchial epithelium of KD patients [24], and the prominent IgA immune response support the hypothesis that KD is a viral-induced inflammatory disease in genetically susceptible children. However, no causative agent has been found yet. A strain of human coronavirus was proposed as a potential trigger for KD in 2005 [25], but the relationship was then rejected. A specific antigen recognized by the immune response in KD has recently been demonstrated [24], but the identified epitope has yet to be better characterized.

The immunological asset of MIS-C, similarly to KD, features upregulations of IL-1β and IL-18, which are two cytokines belonging to a common immunological pathway. Their release is induced by the activation of Toll-like receptors (TLR) expressed on endothelial cells resulting in NF-kB activation [26]. In contrast, MIS-C does not show an involvement of the IL-17A pathway [18,27], whose activation is implicated in KD pathogenesis. 

KD patients also show increased levels of T-helper type 17 cells that are not observed in MIS-C. This leads to hypothesizing IL-17A and T-helper type 17 cells as the distinguishing feature between the two diseases [27]. 

The evidence of IL-1 pathway involvement leads physicians to use Anakinra, an antagonist of IL-1 receptor in this selected case, with a satisfactory outcome [27,28,29]. 

Italy was one of the first Western countries hit by the pandemic, and Emilia-Romagna was one of the high-incidence areas during all the outbreaks. The present study aimed to define the laboratory and immunological profile of MIS-C, its cardiac manifestations, and its evolution in comparison to KD in the same metropolitan area during the same time period in children exposed to the same environmental agents and restriction measures.

## 2. Materials and Methods

A cross-sectional prospective multicenter study was conducted including all consecutive patients aged from 0 to 17 years diagnosed with KD and MIS-C from April 2020 to April 2021 in two pediatric departments in Bologna (Department of Pediatrics, Istituto di Ricovero e Cura a Carattere Scientifico St. Orsola Polyclinic, University of Bologna and Department of Pediatrics, Maggiore Hospital). 

KD diagnoses were made according to 2017 American Heart Association (AHA) Guidelines [30]. 

MIS-C was defined according to WHO criteria, including clinical, laboratory, and microbiological features, in patients with evidence of SARS-CoV-2 infection or likely contact with confirmed cases [12]. 

Children were admitted to the ICU if one or more of the following were present: shock/hypotension not responding to fluid replenishment, threatening organ disease, or concern for rapid progression.

For both KD and MIS-C, the onset of illness was defined as the first day of fever. 

“Acute phase” was identified as the first 10 days from onset of fever, “subacute phase” was identified as the period ranging from the 11th day to the 20th day after the onset of fever. 

Clinical features, laboratory values, and echocardiogram parameters were collected from each patient. 

Laboratory tests performed in patients were part of a specific protocol established prior to each patient’s hospitalization. Tests were performed before any treatment and included complete blood cell count (white blood cells (WBC), red blood cell count, (WBC)), C-reactive protein (CRP), erythrocyte sedimentation rate (ESR), ferritin, fibrinogen, triglycerides, alanine-aminotransferase (ALT), aspartate-aminotransferase (AST), fibrinogen, ferritin, albumin, sodium, creatine kinase (CK); tumor necrosis factor alpha (TNF-alpha), interleukin-8 (IL 8), interleukin-12p70 (IL 12p70), interleukin-10 (IL-10), and interleukin-6 (IL-6). Myocardial injury was evaluated by Troponin-I (cTnI) and B-type natriuretic peptide (BNP). Immunoglobulin levels (IgG, IgA, IgM) were evaluated through the turbidimetric method; lymphocyte immunophenotyping CD3+ (PAN-T), CD3+CD4+ (T-helper cells), CD3+CD8+ (T-cytotoxic cells), CD19+ (PAN-B), and CD16+56+ (NK-cells) was completed through multiparametric flow cytometry. 

Repeated transthoracic echocardiograms (TTE) were performed in all patients during the acute and subacute phases, and the following data were collected: diameters of left ventricle, systolic function expressed by ejection fraction (EF) measured by Simpson’s rule, mitral and aortic valve function, and the presence of pericardial effusion. The diameters of coronary arteries were firstly measured in mm; then, they were indexed for body surface area and expressed as z-score according to the method of Dallaire and Dahdah [30,31]). Left ventricular dysfunction was defined by EF < 55%. 

The degree of coronary involvement was expressed as z-score and stratified according to the 2017 AHA criteria: z-score < 2, normal; 2 to 2.5, dilation; 2.5 to 5, small aneurysm; 5 to 10 (and absolute size < 8 mm), medium aneurysm; ≥10 (or absolute size ≥ 8 mm), large/giant aneurysm. 

TTEs were performed at onset and repeated in case of cardiac involvement during the in-hospital stay according to the degree of the injury and, following discharge, weekly for the first month, then monthly for 3 months, ultimately after 6 and 12 months from the diagnosis. 

Patients diagnosed with KD received immunoglobulins (IVIG) at 2 g/Kg in a single infusion within the tenth day from onset, aspirin at 30–50 mg/Kg/day, subsequently reduced to 3–5 mg/Kg/day after > 48 h without fever [30]. 

Patients diagnosed with MIS-C received immunoglobulins (IVIG) at 2 g/Kg in a single infusion within the tenth day, methylprednisolone at 2 mg/kg/day in case of shock and/or threatening organ disease, and aspirin at 3–5 mg/Kg/day [32]. Patients with an elevation of D-Dimer more than 5 times above the normal values received enoxaparin sodium at 100 U/Kg twice a day [33]. 

All patients were tested for COVID by detection of SARS-CoV-2 RNA in naso-pharyngeal swab specimens by a real-time RT-PCR system (Simplexa™ COVID-19 Direct assay, DiaSorin, Cypress, CA, USA). The assay targets two different regions of the SARS-CoV-2 genome, ORF1ab, and S gene. 

The quantitative determination of antibodies to the SARS-CoV-2 spike (S) protein receptor binding domain (RBD) in human serum was performed using Elecsys^®^ Anti-SARS-CoV-2 S immunoassay, Roche Diagnostics (Rotkreuz, Switzerland). The assay uses a recombinant protein representing the RBD of the S antigen in a double-antigen sandwich assay format, which favors the detection of high-affinity antibodies against SARS-CoV-2. 

The study was approved by the Hospital’s Investigational Review Board. Written informed consent was collected by parents/legal tutors for each participant. 

The study was conducted according to the guidelines of the Declaration of Helsinki and approved by the Ethics Committee of Area Vasta Emilia Centro (protocol numbers: 98/2016/O/Sper and 178/2021/Sper/AOUBo). Patients were only included after giving their or their parental informed consent. 

### Statistical Analysis

Continuous data are presented as mean ± standard deviation (SD). We tested the normality for each variable through the Kolmogorov–Smirnov test. For categorical variables, the percentage of patients in each category was calculated and compared with Chi-square or Fisher’s exact test when appropriate. The two groups were compared running an Independent-Samples T-Test. Levene’s test was used to assess the equality of variances for the considered variables. *p* < 0.05 was considered statistically significant. The study analysis was performed using SPSS V26 for Macintosh.

## 3. Results

Demographic data and clinical features are displayed in Table 1.

Twenty-four patients were diagnosed with MIS-C (14 (58.33%) boys; 21 (87.5%) Caucasian): three patients were diagnosed during the first outbreak (from April 2020 to July 2020), while the remaining 21 were diagnosed during the second outbreak (from October 2020 to January 2021) and the third outbreak (from February 2021 to May 2021). 

Median age was 82 months (First Interquartile Range (IQR1): 59.5, Third Interquartile Range (IQR3): 108); median in-hospital stay was 9 days (IQR1:7, IQR3:11). 

The laboratory values of the acute and subacute phases are expressed in Table 2. Lymphocyte subsets of MIS-C and KD patients during the acute phase are reported in Table 3.

Nine patients (37.5%) presented a positive molecular naso-pharyngeal swab for SARS-CoV-2. When performed, serology for SARS-CoV-2 resulted always positive (19/19 patients).

Cardiac features are reported in Table A1 in Appendix A. At a median time of 5 days (IQR1: 4, IQR3: 6) from the onset of fever, 16 patients (66.67%) developed cardiac involvement: mean cTnI values were 684.02 ± 2090.25 ng/mL (normal values < 19.8 ng/mL) and BNP values were 878.38 ± 1314.63 pg/mL (normal values < 100 pg/mL). Echocardiographic showed left ventricular dysfunction in 11 cases (45.83%) with EF median values of 40 (IQR1: 40-IQR3: 49), valvular dysfunction in 10 (41.66%), and CALs in 11 (45.83%): seven dilations (29.13%) and four aneurysms (16.66%, mean z-score 4.49 + 1.72). New onset arrhythmias were detected in eight (33.33%) (Appendix A, Table A1), only two of whom (8.33%) were still detectable in the subacute phase. 

All patients but one received IVIG and steroids at a median time of 5 days from onset of fever (IQR1:4; IQR3:6).

One patient received infliximab because of persistence of fever, conjunctival hyperemia, and the development of CALs after the first dose of IVIG and steroids, with subsequent rapid defervescence. 

Thirteen patients (13/24, 54.13%) required inotropic support: dopamine was used in 8/13 patients (61.54%), milrinone in 3/13 (23.08%), epinephrine in 2/13 (15.38%), and dobutamine in 2/13 (15.38%). The median duration of inotropic therapy was 4 days (IQR1: 4; IQR3: 5). 

Seventeen patients (70.83%) received enoxaparin sodium during the hospitalization. 

At a median time of 6 days (IQR1: 5, IQR3: 7) after administration of therapy, cardiac markers and left ventricular dysfunction normalized in all patients. 

During the same time-period, 15 patients were diagnosed with KD. Demographic data are expressed in Table 1; themedian in-hospital stay was 8 days (IQR1:7, IQR3:11). Seven (46.66%) were males, and 14 (93.3%) were Caucasian. 

All KD children had negative molecular naso-pharyngeal swab and serology for SARS-CoV-2. 

Two patients were IVIG non-responders and received a supplementary dose of IVIG; four patients (26.67%) received initial glucocorticoids. All patients showed normal cardiac function during acute (median time of 6 days, IQR1: 5, IQR3: 10) and subacute stages; seven patients (46.67%) developed coronary anomalies: three aneurysms (42.86%), and four dilations (57.14%). Aneurysms persisted at a median time of 6 days from the administration of therapy (IQR1:5; IQR3: 14.25) and 15.5 days after onset (IQR1:11.5, IQR3: 23.75), while the four coronary dilations regressed. At a median time of 46 days from onset (IQR1:41, IQR3: 61) and of 36 days from the administration of therapy (IQR1:24, Q3: 53), aneurysm persisted in one patient (33.3%) and regressed to dilations only in the remaining two (66.6%).

We compared demographics and clinical features (see Table 1) and laboratory data between MIS-C and KD patients (see Table 1 and Table 2, respectively). 

MIS-C patients were significantly older than KD patients (75 months vs. 28 months, *p* = 0.001). 

Fever lasted significantly longer (*p* = 0.0024) in KD patients (median: 10 days) than in MIS-C patients (median: 6 days).

KD patients presented more frequently skin rash (*p* = 0.038), changes of the lips and oral cavity (*p* = 0.008), and cervical lymphadenopathy (*p* = 0.008) than MIS-C patients. 

On the contrary, MIS-C patients reported more frequently abdominal symptoms such as abdominal pain, nausea, vomiting and diarrhea (*p* = 0.004), andrespiratory symptoms (*p* = 0.004). Four MIS-C patients (16.66%) underwent appendicectomy because of acute abdomen-like clinical presentation. Histology showed inflammatory infiltrates and angiogenesis involving the wall of several arteries and veins in the periappendiceal fat. The composition of this perivascular transmural infiltrate reflected the difference in clinical severity.

**Figure 1:**
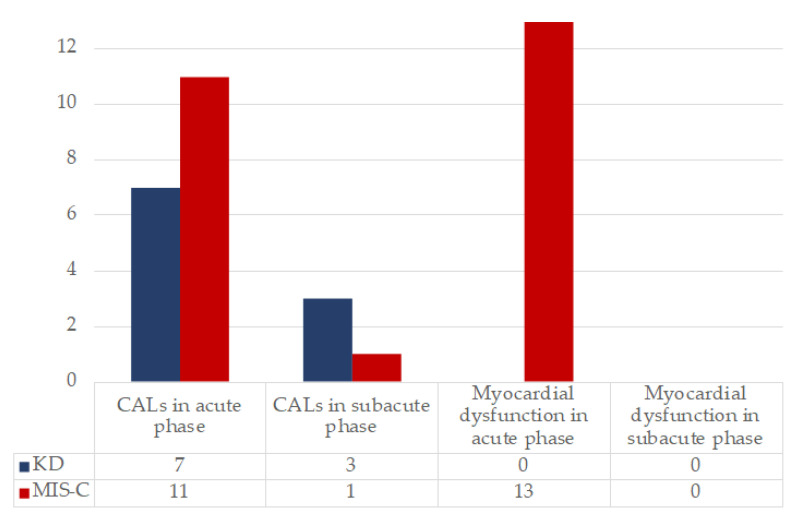
Evolution of cardiac involvement during acute and subacute phase in KD and MIS-C. On the *y* axis, the number of patients presenting the specific feature is reported.

Cardiac dysfunction was significantly more frequent in patients diagnosed with MIS-C (*p* = 0.001). The incidence of CALs was similar between the two groups during the acute phase. CALs regressed soon after treatment in 10/11 MIS-C, while they were still detectable during the subacute stage in 3/7 (42.8%) and during convalescent stage in 1/7 (14.3%) KD patients with a significant difference between the two groups (*p* = 0.034). Mean TnI values were significantly higher in MIS-C than in KD (*p* = 0.026) (Table 2). Coronary and non-coronary cardiac findings are shown in Figure 1. Characteristics of CALs and other cardiac features are shown in Appendix A, Table A1.

Among the cytokines tested, only IL-8 values were significantly higher in MISC-C patients (*p* = 0.021) (Table 2). Among the other values, AST, ALT, fibrinogen, albumin, sodium, and potassium levels were similar in both groups. 

Peripheral lymphocyte subsets during the acute phase did not show any significant difference between MIS-C and KD patients (Table 3). 

## 4. Discussion

One year after the first reports of MIS-C, the underlying mechanism of pathogenesis remains unclear. Since both KD and MIS-C are systemic vasculitides sharing clinical, laboratory, and cardiac involvement phenotype, we hypothesized that the cytokine profile and lymphocyte subsets could define a peculiar pattern useful for distinguishing the two diseases. We also hypothesized that the characteristics of cardiac injury could help understand the pathophysiology of the tissue damage. Most of the diagnoses of MIS-C in our center were made during the second and third Italian outbreak of COVID-19, after October 2020, when the alpha variant (named the English variant) was the most prevalent. As expected, according to the post-acute nature of the disease, most of the MIS-C patients’ naso-pharyngeal swabs were negative for SARS-CoV-2 on admission, so viral typing was not possible. Despite this, all MIS-C patients tested presented SARS-COV-2 IgG antibodies: in patients without PCR positivity, IgG could reflect the previous viral contact because of effective virus clearance by immune system. 

Our data confirm that MIS-C patients are older than KD, accordingly with the literature [17,18]. Despite a severe presentation including myocardial injury and the need for ICU admission more frequently than KD patients, the vast majority of MIS-C patients rapidly and completely responded to immunomodulatory treatment within 6 days. Notably, immunomodulatory treatment was intensified with steroids in all children with MIS-C with one exception. The indications were shock or signs of threatening organ disease, as recommended by the American College of Rheumatology [32].

More than 60% of MISC patients presented initial cardiac damage, in most cases with left ventricular dysfunction requiring inotropic support and extremely high troponin levels. This finding together with the improvement of myocardial function and markers of cardiac injury soon after immunomodulatory treatment could suggest that cardiac damage is likely to be due to cytokine storm-induced myocardial stunning and arterial vasoplegia rather than direct cytotoxic injury in MIS-C. In this scenario, an early control of inflammation may limit the disease’s severity. Our hypothesis appears to be supported by early cardiac MRI and speckle tracking echocardiography abnormalities [34] suggesting a post-viral immune-mediated myocarditis-like pathogenesis rather than a virus-mediated process [35]. 

The incidence of coronary anomalies is similar between the two diseases during the acute stage. Anomalies regress in most cases of MIS-C soon after treatment, while they tend to persist in KD: during the subacute stage, CALs were documented in more than 40% of KD but only in 12.5% of MIS-C patients. The differences in severity at presentation and in coronary outcome reasonably support the hypothesis that the underlying pathogenic mechanisms are different in MIS-C and KD. It is worth noting that MIS-C patients received a more intense immunomodulatory treatment that could have affected the coronary outcome.

The effectiveness of the immunomodulatory response and the rapid restore of ventricular function support the role of an aberrant cytokine-driven inflammation in the pathogenetic mechanism of myocardial damage rather than a direct viral cytotoxic injury. Long-term cardiac MRI and the comparison with viral myocarditis outcomes are mandatory to confirm our speculations. Even though long-term cardiac outcome in patients suffering from MIS-C seems to be favorable, a follow-up program is recommended for these patients. 

MIS-C patients showed higher levels of IL-8 during the acute stage of disease compared to KD patients. This led us to hypothesize a role for IL-8 in the pathogenesis of MIS-C. IL-8 belongs to the group of pro-inflammatory cytokines, working as a neutrophils chemotactic factor during inflammation. An elevation of IL8, together with IL-10 and IL-6 has already been described in MIS-C [36,37], even though the role of these cytokines has not been fully clarified yet.

Among its functions, IL-8 contributes to induce endothelial permeability by inducing the expression of integrins, enabling neutrophils to pass through vessels to reach inflammation sites [38] and preventing clot formation [39].

It is also implied in coagulation that high IL-8 values lead to higher risk for the development of venous thrombosis, and it was recently suggested that IL-8 elevation may potentially indicate a higher risk for thrombosis development.

Some authors discussed the role played by coagulation in MIS-C, especially in producing thrombotic microangiopathy [40,41], even though its involvement is still far from being fully understood.

IL-8 could also be implicated in the endothelitis developing during MIS-C. The activation product of the terminal complement cascade, soluble C5b-9, previously associated with microangiopathy, was found to be high in MIS-C and significantly correlated with IL-8, IL-6, and TNF-alpha [42].

Our findings could suggest that endothelial dysfunction in MIS-C mediated by distal complement system is more pronounced than in KD.

IL-8 plays a role in COVID-19 manifestations: high serum IL-8 levels at the onset are independent predictors of patient survival [43] and are associated with the duration of illness [44], so it represents a potential target for treatment [45,46].

Despite far from being completely understood, KD is thought to develop in genetically predisposed children exposed to an environmental agent, probably a virus, with imbalance in the immune system characterized by increased Th1/Th17-related immunity and a Treg/Th17 imbalance, which is followed by increased production of inflammatory cytokines such as TNF-α, IL-1, IL-2, IL-6, IL-8, MCP-1, and GM-CSF, leading to aberrant activation of neutrophils and macrophages.

In our cohorts, apart from IL-8, the other tested cytokines and lymphocytes subpopulations were comparable between MIS-C and KD. This finding is in contrast to the data described in larger series potentially due to the small sample size of our study 

Despite not reaching a statistical significance, IL-10 was higher in MIS-C, while IL-1 beta was higher in KD.

IL-10 is the principal immunoregulatory cytokine acting to prevent a disproportioned immune activation leading to self-damage of host tissues: by binding its receptor on leucocytes, it induces the activation of the Jak-STAT signaling pathway with the effect of negatively regulating inflammation [47]. IL-10 exerts its effect through a paracrine activity in experimental models [48] as confirmed by a positive therapeutic effect in autoinflammatory diseases only when administered locally [49]. If administered systemically, on the contrary, it induces a paradoxical pro-inflammatory effect [50].

During viral infections, IL-10 is released by dendritic cells during the very first phases of immune response, right after the stimulation of antigen-presenting cells by viral pathogens-associated molecular patterns and danger-associated molecular patterns with the purpose of preventing an excessive immune response and consequent tissue damage [47]. Secondly, IL-10 enhances Natural Killer cells survival, inducing a virtuous cycle in which NK cells themselves start producing IL-10, expanding the control of innate immune response. Lately during the antiviral response, IL-10 production becomes dependent on CD8+ and CD4+ cell [47,51,52,53].

The huge release of IL-10 modulates the function of dendritic cells and antigen-presenting cells in general, limiting antigen exposure to T lymphocytes, reducing the differentiation of naïve T cells into Th1 cells, and eventually stopping T lymphocytes responses. 

During persistent central nervous system infection sustained by coronaviruses neurotropic strains, IL-10 mRNA is upregulated [51], and IL-10 deficiency is associated to broader virus-induced CNS damage [52].

IL-10 has a biphasic action in virus clearance by adaptive immunity, CD8+ T cells being the main producer when viral load is still high and subsequently replaced by CD4+ T cells after viral load is reduced, in particular by CD4+ CD25+ Treg cells [51].

Since MIS-C develops up to 6 weeks after SARS-CoV-2 infection, it is reasonable to believe that the higher IL-10 levels observed in our population are to be attributed to a late IL-10 production by CD4+ T cells. This may be interpreted as a sign of SARS-CoV-2 persistence in MIS-C patients leading to a chronic antigen stimulation.

IL-10 could also play a role in modulating endothelial function: among other cytokines, it significantly increases in adults developing massive pulmonary embolism compared to patients with lower extension of disease [39]. Models of IL-10-deficient mice found reduced vascular relaxation, stiffer arteries, cardiomegaly, and heart dysfunction compared to wild-type murine models, suggesting a crucial role of this cytokine in endothelial function [54,55,56].

Previous studies on myocardial dysfunction during sepsis underlined the role played by the cytokine storm, especially by TNF-α and IL-1β in inducing a precocious decrease in myocardial contractility [57]. IL-6 was recognized as responsible for myocardial stunning both in animal models and in humans [58]. In vitro studies suggested that cytokines’ depressing effect on myocardial function appears to be related to a disruption in myocardial excitation–contraction coupling [59].

In our cohort of MIS-C patients, IL-6 values were elevated, while TNF-alpha, IL-1-beta, and IL-12p70 values were normal. 

As stated above, MIS-C patients were significantly older than KD patients. The marked difference in age between the two groups is still far from being fully understood; nevertheless, it appears to be a potentially distinguishing feature between the two patterns. 

MIS-C and KD are commonly characterized by a disproportionate reaction of immune system to a trigger. While an MIS-C trigger has clearly been recognized as SARS-CoV-2, the KD trigger, despite being largely debated, has not been found yet [60,61,62]. 

The state of the art still does not allow us to clearly establish whether MIS-C and KD are two distinguishable independent diseases or two sides of a coin. The marked difference in age must be kept in mind when trying to analyze the differences between MIS-C and KD. Viral-triggered immune response in older patients may lead to the peculiar clinical pattern that results in MIS-C, while KD may result from a secondary hyperinflammatory response to a worldwide endemic virus, toward which older children have acquired natural immunization, which is absent for the new SARS-CoV-2. 

MIS-C and KD patients received different treatment regimens. All MIS-C patients received high-dose steroids and IVIG, while the vast majority of KD patients only received IVIG. Our data do not allow us to exclude that the more favorable evolution of MIS-C may be related to the additional steroid treatment. Indeed, by acting on the earliest stages of the arteries’ remodeling process, steroids might explain the more favorable coronary outcome of MIS-C patients compared to KD patients. 

In total, 12.5% of MIS-C patients from our cohort showed overlapping clinical features with KD by satisfying the diagnostic criteria of KD according to 2017 AHA guidelines. Immunological features, including cytokine levels, of the three patients were very similar to those of other MIS-C patients. This finding is most likely biased by the very small number of patients (3); indeed, it would be very interesting to collect more robust immunological data from patients with symptoms overlapping between the two diseases. In this context, clinicians should also be aware that KD can occur in children who previously experienced a SARS-CoV-2 infection.

Our study has both strengths and limitations: notably, all laboratory tests, including cytokines profile and lymphocyte subpopulations, were collected before any immunomodulatory treatment in both cohorts of patients. Moreover, the diagnoses of MIS-C and KD were made during the same time period in the same geographic area; thus, children were potentially exposed to the same environmental factors, including infectious agents, as well the lock-down restrictions. On the other side, the size of the cohort was modest but large enough to observe statistical differences. 

## 5. Conclusions

In summary, the different cytokines profile of MIS-C compared to KD suggests a role of IL-8 in the pathogenesis of the disease by inducing endothelial dysfunction. IL-10 despite being increased was not significantly higher in MIS-C than in KD: a larger number of cases may contribute to a statistical difference amongst IL-10 values. Immunological response was similar to KD, potentially reflecting a similar lymphocytes activation. Despite the dramatic onset characterized by frequent and severe cardiac involvement, laboratory markers and echocardiographic features of myocardial injury regress to normal soon after treatment. The rapid cardiac response to immunomodulatory treatment in terms of markers of myocardial damage and ventricular systolic function supports a cytokines-driven mechanism rather than a direct cytotoxic virus-induced injury. In addition, coronary lesions tend to regress after treatment in most MIS-C patients, while they tend to persist in up to 40% of KD cases. The differently-mediated endothelial dysfunction could explain the different evolution. 

Further studies are needed to confirm our main findings.

## Figures and Tables

**Table 1:** Comparison between demographics and clinical features of MIS-C and KD patients.

Demographic Data		MIS-C	KD	*p*-Value
Ethnicity, n (%)	Caucasian	21 (87.50%)	14 (93.3%)	n.s.
	Asiatic	1 (4.16%)	0 (0%)	
	Hispanic	0 (0%)	0 (0%)	
	Others	0 (0%)	1 (6.6%)	
	African	2 (8.33%)	0 (0%)	
Sex, n (%)	Male	14 (58.33%)	7 (46.66%)	n.s.
	Female	10 (41.67%)	8 (53.33%)	
Age in months, median (IQR1-IQR3)		82 (59.5–108)	27 (22.5–42.5)	0.00
In-hospital stay, median (IQR1-IQR3)		9 (7.25–11)	8 (7–11)	n.s.
ICU admission (%)		11 (45.83%)	0 (0%)	0.003
Clinical features, n (%)	Bilateral bulbar conjunctival injection	13 (54.17%)	12 (80%)	n.s.
	Erythema and edema of the hands and feet	8 (33.3%)	7 (46.66%)	n.s.
	Rash	12 (50%)	13 (86.66%)	0.038
	Erythema and cracking of lips/strawberry tongue/erythema of oral and pharyngeal mucosa	10 (41.67%)	13 (86.66%)	0.008
	Cervical lymphadenopathy	3 (12.5%)	12 (80%)	0.000
	Abdominal symptoms (abdominal pain, diarrhea)	21 (87.5%)	6 (40%)	0.004
	Respiratory symptoms (cough, respiratory failure)	6 (25%)	0 (0%)	n.s.
	Hypotension	8 (33.33%)	0 (0%)	0.017

**Table 2:** Laboratory values of MIS-C and KD patients during the acute stage. SD stands for standard deviation.

Laboratory Value	Normal Values	Group	Mean (± SD)	*p*-Value
WBC (/mmc)	4800–12,000	KD	13,271.80 (±5831.09)	n.s.
		MIS-C	10,899.43 (±6356.01)	
Lymphocytes (%)	25.0–55.0	KD	20.33 (±12.03)	n.s.
		MIS-C	15.63 (±12.26)	
Neutrophils (%)	28.0–71.0	KD	71.19 (±12.94)	n.s.
		MIS-C	74.21 (±19.93)	
Lymphocytes (/mmc)	1800–7000	KD	1950.02 (±1406.84)	n.s.
		MIS-C	1373.32 (±1252.9)	
Neutrophils (/mmc)	1700–8500	KD	9452.43 (±5678.6)	n.s.
		MIS-C	7766.51 (±6317.94)	
Platelets (/mmc)	180,000–415,000	KD	326,016.93 (±144,952.74)	n.s.
		MIS-C	238,833.33 (±144,952.43)	
ESR (mm/h)	<11	KD	59.45 (±22.89)	n.s.
		MIS-C	41.25 (±17.78)	
CRP (mg/dL)	<0.5	KD	11.92 (±6.90)	n.s.
		MIS-C	18.07 (±9.53)	
Procalcitonin (ng/mL)	<0.1	KD	7.86 (±14.16)	n.s.
		MIS-C	21.61 (±30.72)	
IL-6 (pg/mL)	<5.9	KD	146.00 (±135.85)	n.s.
		MIS-C	140.84 (±161.43)	
IL-10 (pg/mL)	<5.3 (adults)	KD	4.78 (±3.67)	n.s.
		MIS-C	29.26 (±61.60)	
IL-8 (pg/mL)	<70 (adults)	KD	52.11 (±103.19)	0.021
		MIS-C	91.85 (±129.82)	
TNF-alpha (pg/mL)	<8.1 (adults)	KD	2.00 (±5.29)	n.s.
		MIS-C	1.11 (±1.54)	
IL-1 beta (pg/mL)	<6.7 (adults)	KD	4.57 (±10.36)	n.s.
		MIS-C	0.83 (±0.75)	
IL-12p70 (pg/mL)	<4.7 (adults)	KD	0.43 (±0.53)	n.s.
		MIS-C	0.50 (±0.58)	
D-Dimer (mg/L)	<0.5	KD	2.18 (±1.48)	n.s.
		MIS-C	3.60 (±2.39)	
Ferritin (ng/mL)	25 –335	KD	163.60 (±58.79)	n.s.
		MIS-C	449.47 (±406.94)	
BNP (pg/mL)	<100	KD	235.25 (±221.02)	n.s.
		MIS-C	878.38 (±1314.63)	
TnI (ng/L)	<19.8	KD	2.67 (±2.87)	0.026
		MIS-C	684.02 (±2090.25)	

**Table 3:** Comparison of lymphocytes subpopulations of MIS-C and KD patients during the acute phase. Values are expressed as median (IQR1-IQR3).

	MIS-C	KD	*p*-Value
CD3+(PAN T)%	54 (49–59.5)	65 (54.25–67.5)	n.s.
CD3+/CD4+%	32 (27.5–40.5)	34.50 (32.25–39.75)	n.s.
CD3+/CD8+%	20 (17.5–24)	20 (14.5–21.75)	n.s.
CD4+/CD8+	1.50 (1.26–2.13)	2.10 (1.71–2.54)	n.s.
CD56+/CD16+/CD3-(NK)%	8 (5.5–13)	6 (6–16)	n.s.
CD19 (PAN B) %	33.56 (25.67–37.96)	25.65 (20.03–27.45)	n.s.

## Appendix A

**Table A1:** Features of cardiac involvement in MIS-C and KD.

		MIS-C	KD
CALs, n (%)	Dilation (2 < z-score < 2.5), n (%)	7 (29.13%)	3 (20%)
	Aneurysm (z-score > 2.5), n (%)	4 (16.66%)	3 (20%)
TTE findings	Pericardial Effusion, n (%)	4 (16.66%)	3 (20%)
	Mitral/Aortic valve regurgitation, n (%)	10 (41.66%)	3 (20%)
	EF < 55%, n (%)	11 (45.83%)	0
	Valvular regurgitation, n (%)	10 (41.66%)	3 (20%)
	Pericardial effusion	4 (16.66%)	3 (20%)
	Median EF (IQR1-IQR3)	58 (48–64.5)	65 (61.5–68.5)
Arrhythmias, n (%)	Nonspecific ST-T repolarization abnormalities, n (%)	3 (12.5%)	1 (6.66%)
	First-degree atrioventricular block, n (%)	3 (12.5%)	0
	QT prolongation, n (%)	1 (4.17%)	0
	Incomplete right bundle branch block, n (%)	1 (4.17%)	0
